# Video capture virtual reality as a flexible and effective rehabilitation tool

**DOI:** 10.1186/1743-0003-1-12

**Published:** 2004-12-20

**Authors:** Patrice L Weiss, Debbie Rand, Noomi Katz, Rachel Kizony

**Affiliations:** 1 Dept. of Occupational Therapy, University of Haifa, Israel; 2 School of Occupational Therapy, Hadassah-Hebrew University, Israel; 3 Dept. of Occupational Therapy, Chaim Sheba Medical Center, Israel

## Abstract

Video capture virtual reality (VR) uses a video camera and software to track movement in a single plane without the need to place markers on specific bodily locations. The user's image is thereby embedded within a simulated environment such that it is possible to interact with animated graphics in a completely natural manner. Although this technology first became available more than 25 years ago, it is only within the past five years that it has been applied in rehabilitation. The objective of this article is to describe the way this technology works, to review its assets relative to other VR platforms, and to provide an overview of some of the major studies that have evaluated the use of video capture technologies for rehabilitation.

## Introduction

Two major goals of rehabilitation are the enhancement of functional ability and the realization of greater participation in community life. These goals are achieved by intensive intervention aimed at improving sensory, motor, cognitive and higher level-cognitive functions on the one hand, and practice in everyday activities and occupations to increase participation on the other hand [1,2]. Intervention is based primarily on the performance of rote exercises and/or of different types of purposeful activities and occupations [3,4]. The client's cognitive and motor abilities are assessed throughout the intervention period so that therapy may be continually adjusted to the client's needs. For many injuries and disabilities, the rehabilitation process is long and arduous, and clinicians face the challenge of identifying a variety of appealing, meaningful and motivating intervention tasks that may be adapted and graded to facilitate this process. Clinicians also require outcomes that may be measured accurately. Virtual reality-based therapy, one of the most innovative and promising recent developments in rehabilitation technology, appears to provide an answer to this challenge. Indeed, it is anticipated that virtual reality (VR) will have a considerable impact on rehabilitation over the next ten years [5].

Virtual reality typically refers to the use of interactive simulations created with computer hardware and software to present users with opportunities to engage in environments that appear to be and feel similar to real world objects and events [6-8]. Users interact with displayed images, move and manipulate virtual objects, and perform other actions in a way that attempts to "immerse" them within the simulated environment thereby engendering a feeling of presence in the virtual world [9,10].

The objective of this article is to briefly describe the use of VR in rehabilitation, and then emphasize the unique attributes of the video capture VR to rehabilitation, including an overview of some of the major studies that have evaluated the use of this technology for rehabilitation.

## Virtual reality applied to rehabilitation

Virtual reality has a number of well-known assets, which make it highly suitable as a rehabilitation intervention tool [11]. These assets include the opportunity for experiential, active learning and the ability to objectively measure behavior in challenging but safe and ecologically-valid environments while maintaining strict experimental control over stimulus delivery and measurement. VR also provides the capacity to individualize treatment needs, while gradually increasing the complexity of tasks and decreasing the support provided by the clinician [5,12].

During the mid to late 1990s, virtual reality technologies first began to be developed and studied as potential tools for rehabilitation assessment and treatment intervention [7]. The list of applications is long and diverse, and only several examples are provided here. VR has been used as a medium for the assessment and rehabilitation of cognitive and metacognitive processes, such as visual perception, attention, memory, sequencing and executive functioning [13]. Rizzo and colleagues [14,15] developed a Virtual Classroom for the assessment and training of attention in children with Attention Deficits Hyperactive Disorder. Piron, et al. [16] used a virtual environment to train reaching movements, Broeren, et al. [17] used a haptic device for the assessment and training of motor coordination, and Jack et al. [18] and Merians, et al. [19] have developed a force feedback glove to improve hand strength and a joint position glove to improve the range of motion and speed of hand movement. The studies cited above share a common goal of using virtual reality to construct a simulated environment that aimed to facilitate the client's motor, cognitive or metacognitive abilities in order to improve functional ability. In some cases, the applications take advantage of the ability to adapt virtual environment to simulate real life activities such as meal preparation [20] or crossing a street [21-25]. The ultimate goal of such applications is to enable clients to become able to participate in their own real environments in a more independent manner. Attempting to achieve similar results via conventional therapy when clinicians and clients must deal with real world settings (e.g., a visit to a real supermarket) is fraught with difficulty. In contrast, virtual environments may be adapted with relative ease to the needs and characteristics of the clients under care.

Given the variety of VR platforms and the diverse clinical populations that may benefit from VR-based intervention, it is helpful to view the VR experience as a multidimensional model that appears to be influenced by many parameters. A conceptual model was developed within the context of terminology established by the International Classification of Functioning, Disability and Health (ICF) [2] and the rehabilitation process [25,26]. This model helps to identify the clinical rationale underlying the use of virtual reality as an intervention tool in rehabilitation as well as to design research to investigate its efficacy for achieving improved performance in the real world. The process of using VR in rehabilitation is modeled via three nested circles, the inner "Interaction Space", the intermediate "Transfer Phase" and the outer "Real World".

The "Interaction Space" denotes the interaction that occurs when the client performs within the virtual environment, experiencing functional or game-like tasks of varying levels of difficulty, i.e., the activity component according to the ICF terminology. This interaction is influenced by user characteristics, which include personal factors (e.g. age, gender, cultural background), body functions (e.g. cognitive, sensory, motor abilities) and structures (e.g., the parts of the body activated during the task). It is also influenced by the characteristics of VR platform and its underlying technology (e.g. point of view, encumbrance) that presents the virtual environment and the nature and demands of the task to be performed within the virtual environment.

It is during the interaction process that sensations and perceptions related to the virtual experience take place; here the user's sense of presence is established, and the process of assigning meaning to the virtual experience as well as the actual performance of virtual tasks or activities occurs. The sense of presence enables the client to focus on the virtual task, separating himself temporarily from the real world environment. This is an important requirement when motor and, especially, cognitive abilities and skills are trained or restored. The concept of meaning is also thought to be an essential factor that enhances task performance and skills in rehabilitation in general [1,3], and thus also in the VR-based rehabilitation [27]. Environmental factors within the virtual environment may contribute information about issues that facilitate or hinder the client's performance, and may serve as facilitators of performance in the virtual environment leading to improved performance in the real world.

Two outer circles, the "Transfer Phase" and the "Real World" denote the goal of transferring skills and abilities acquired within the "Interaction Space" and eliminating environmental barriers in order to increase participation in the real world (i.e., participation in the natural environment according to the ICF terminology). The "Transfer Phase" may be very rapid and accomplished entirely by the client or may take time and need considerable guidance and mediation from the clinician. The entire process is facilitated by the clinician whose expertise helps to actualize the potential of VR as a rehabilitation tool.

## Virtual reality platforms

Virtual environments are experienced with the aid of special hardware and software for input (transfer of information from the user to the system) and output (transfer of information from the system to the user). The selection of appropriate hardware is important since its characteristics may greatly influence what is taking place in the Interaction Space, i.e., the way users respond (e.g. sense of presence, performance) to a virtual environment [28]. The output to the user generates different levels of immersion, which may be enhanced by different modalities including visual, auditory, haptic, vestibular and olfactory stimuli, although, to date, most VR platforms deliver primarily visual and auditory feedback. Visual information is commonly displayed by head mounted displays (HMD), projection systems, or flat screen, desktop systems of varying size. Input to a virtual environment enables the user to navigate and manipulate objects within it. Input may be achieved via direct methods such as inertial orientation tracker or by video sensing which tracks user movement. Input may also be achieved via activation of computer keyboard keys, a mouse or a joystick or even virtual buttons appearing as part of the environment.

In addition to specialized hardware, application software is also necessary. In recent years, off-the-shelf, ready-for-clinical-use VR software has become available for purchase. However, more frequently, special software development tools are required in order to design and code an interactive simulated environment that will achieve a desired rehabilitation goal. In many cases, innovative intervention ideas may entail customized programming to construct a virtual environment from scratch, using traditional programming languages.

## Video capture VR

Video capture VR consists of a family of camera-based, motion capture platforms that differ substantially from the HMD and desktop platforms in wider use. When using a video-capture VR platform, users stand or sit in a demarcated area viewing a large video screen that displays one of a series of simulated environments. Users see themselves on the screen, in the virtual environment, and their own natural movements entirely direct the progression of the task, i.e., the user's movement is the input. The result is a complete engagement of the user in the simulated task. A single video camera converts the video signal of the user's movements wherein the participant's image is processed on the same plane as screen animation, text, graphics, and sound, which respond in real-time. This process is referred to as "video gesture", i.e., the initiation of changes in a virtual reality environment through video contact. The user's live, on-screen video image responds at exactly the same time to movements, lending an intensified degree of realism to the virtual reality experience. Video capture provides both visual and auditory feedback with the visual cues being most predominant.

Myron Krueger [29] was the first to investigate the potential of video capture technology in the 1970s with his innovative Videoplace installation. This was one of the first platforms that enabled users to interact with graphic objects via movements of their limbs and body, and was used to explore a variety of virtual art forms. The quality of the video image in these applications was relatively primitive, consisting of silhouetted figures. Nevertheless, the immediate response of the virtual environment in real-time to the user's movements presented compelling evidence of the possibility of using this technique for interactive simulation.

**Figure 1:**
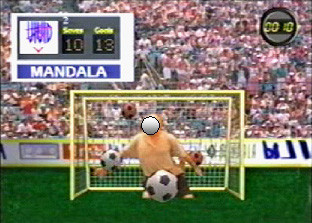
Individual with a stroke performing within the Soccer environment using the VividGroup GX system.

The next major development occurred with the release of VividGroup's Mandala Gesture Extreme (GX) platform  in 1996, together with a suite of interactive, game-type environments. This platform makes use of a chroma key-based setup so that the existing background is subtracted and replaced by a simulated background. GX VR has enjoyed considerable success around the world in numerous entertainment and educational facilities including science museums and entertainment parks. During the past five years it has also begun to be adapted for use in rehabilitation and has generated great interest in clinical settings (see below). GX VR currently offers a wide variety of gaming applications including, Birds & Balls, wherein a user is required to touch balls of different colors; if the touch is "gentle", the balls turn into doves whereas an abrupt touch causes them to burst. In another application, a soccer game, the user sees himself as the goalkeeper whose task it is to prevent balls from entering the goal area (see Figure 1).

In the late 1990s two other commercial companies developed video-capture gaming platforms, Reality Fusion's GameCam and Intel's Me2Cam Virtual Game System [30]. Both of these platforms aimed for the low-cost, general market, relying on inexpensive web camera installations that did not entail the use of the chroma key technique. For reasons that are not clear, Reality Fusion and Intel discontinued their products within the past two years.

**Figure 2:**
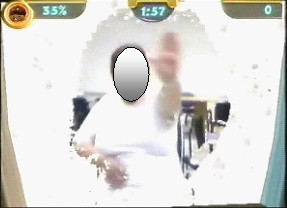
Individual with a stroke performing the Wishy Washy application using the Sony EyeToy system.

Somewhat later, Sony developed its very popular EyeToy application designed to be used with the PlayStation II platform . This is an off-the-shelf, low-cost gaming application, which provides the opportunity to interact with virtual objects that can be displayed on a standard TV monitor [31]. As with the VividGroup's GX platform, the EyeToy displays real-time images of the user. However, it does not require a chroma key blue/green backdrop behind the user nor bright ambient lighting (see Figure 2). This makes for an easier setup of the platform in any location but, on the other hand, it means that the user sees himself manipulating virtual objects within a video image of his own physical surrounding rather than within different virtual environments. An additional difference between the cheaper EyeToy platform and the more expensive GX platform is that the former is capable of recognizing users or objects only when they are in motion. A user who remains stationery does not exist for EyeToy applications. In contrast, the GX VR is responsive to users whether they are in motion or not.

The EyeToy application includes many motivating and competitive environments which may be played by one user or more than one user sequentially in a tournament fashion. With GX VR, two users can compete together simultaneously (e.g., boxing, spinning plates) as well as combine their efforts to create different visual effects without a competitive component (e.g., painting a rainbow, mirror image distortions and popping bubbles).

The potential of these platforms for rehabilitation was readily apparent despite the fact that they were originally developed for entertainment and gaming purposes. Indeed, VividGroups's GX platform was first applied without adaptations within a clinical setting by Cunningham and Krishack [32] who used it to treat elderly patients who were unstable and at high risk for falling. Unfortunately, the inability to grade these platforms to levels suited to patients with severe cognitive or motor impairments initially limited the application of these environments in clinical settings. In order to broaden the potential clinical applications of the platforms, our research group adapted the GX VR platform [33,34]. VividGroup developed, and now also markets, a version of the GX platform, known as IREX (Interactive Rehabilitation EXercise) platform  which enables therapists to adapt levels of difficulty and record performance outcomes [35].

## Characteristics of the Video-Capture Platforms

Video-capture VR differs from other platforms in a number of ways that have great relevance for its use as a tool for rehabilitation evaluation and intervention. Some of these characteristics appear to be advantageous whereas others may limit the utility of video-capture VR.

### Point of View

Video-capture VR provides users with a mirror image view of themselves actively participating within the environment. This contrasts with other VR platforms such as the HMD which provides users with a "first person" point of view, or many desktop platforms in which the user is represented by an avatar. The use of the user's own image has been suggested to add to the realism of the environment and to the sense of presence [10]. It also provides feedback about a client's body posture and quality of movement, comparable to the use of video feedback in conventional rehabilitation during the treatment of certain conditions such as unilateral spatial neglect [36].

### Freedom from encumbrance

The user in video-capture VR does not have to wear or support extraneous devices such as an HMD, glove or markers in order to achieve a substantial intensity of immersion within the virtual environment. This eliminates a source of encumbrance that would likely hinder the motor response of patients with neurological or orthopedic deficits. Although the newer HMDs and stereoscopic glasses are considerably less cumbersome than previous models, little information is available regarding their use by individuals undergoing cognitive or motor rehabilitation.

### Interaction and Control

This characteristic relates to how the user controls objects within the virtual environment. As indicated above, rather than relying on a pointing device or tracker, interaction within video-capture based environments is accomplished in a completely intuitive manner via natural motion of the head, trunk and limbs. Not only is the control of movement more natural, but, in the case of the chroma key GX VR, a "red glove" option (or any object with a distinct color) may be used to restrict system response to one or more body parts as deemed suitable for the attainment of specified therapeutic goals. For example, when it is appropriate to have the intervention directed in a more precise manner, a client may be required to repel projected balls via a specific body part (e.g., by the hand when wearing a red glove or by the head when wearing a red hat). Or, when intervention is more global, the client will not use the red glove option and thus be able to respond with any part of the body. The ability to direct a client's motor response to be either specific or global makes it possible to train diverse motor abilities such as the range of motion of different limbs and whole body balance training.

### Feedback

A limitation of currently available video capture platforms is the reliance on visual and auditory feedback and the absence of a haptic interface that would provide participants with real-time indications of contact with the virtual stimuli. Such feedback could serve as an important addition when used in therapy since the balls, for example, could be rendered to appear to have progressively greater mass, making the task more or less difficult. It would also add an additional element of realism to the gaming experience, and ensure that feedback to participants was more realistic. This could be accomplished to some degree via a quasi-haptic effect that might use vibration to simulate a true haptic interface (A.A. Rizzo, personal communication). For example, small buzzers may be affixed to the tips of the digits. Touching a virtual ball in the Vivid GX Birds & Balls application would generate a low amplitude, high frequency "buzz". In contrast, repelling a larger ball in the Soccer application would generate a high amplitude, low frequency "buzz".

### User position

Video-capture VR may be implemented while users stand, sit, or even walk on a treadmill. For example, the same environment may thus be suitable for training standing balance of a patient who had a stroke, sitting balance of an individual with an incomplete quadriplegic spinal cord injury, and balance during treadmill locomotion of an individual with a paraplegic spinal cord injury.

### Multiple users

Moreover, one or more users may participate within the same environment. In some applications, the ability to have two "rival" users interact simultaneously within the same game or task adds an element of competitiveness that may be motivating. Of greater importance is the ability of the therapist to support a client or use handling techniques in order to facilitate active movement while the client interacts with the virtual stimuli. The therapist can be concealed behind the client in order not to be seen in the VE, or can join the client within the virtual environment.

### Two-dimensional motion plane

Another limitation of the currently available video capture VR platforms is that they may be operated with only one camera. This means that all tasks must be performed within a single plane. In the case of the typical coronal plane setup where the camera is positioned to face the user, any functional movement that takes place in the sagittal or transverse planes is disregarded. Virtual scenarios must therefore be carefully designed such that a meaningful task can be performed despite the restriction to uniplanar movement. Moreover, care must be taken when analyzing the kinematic trajectories since any out-of-plane motion will not be recorded. It is encouraging to note that three dimensional, functional environments will likely soon become available (I. Cohen and A.A. Rizzo, personal communication).

## Applications of video-capture VR in rehabilitation

Although video-capture platforms have only begun to be used for rehabilitation applications within the last five years, there are already results from a number of research groups who have studied its utility with different patient populations. In this section we highlight the major studies that provide evidence that this technology appears to be suitable for use in rehabilitation. The evidence concerning participant sense of presence, enjoyment, usability and performance are summarized as reported by studies of single platforms and by studies that compared different VR platforms

### Side effects

None of the studies carried out to date have reported any significant occurrence of cybersickness-type side effects when using video-capture VR. Rand et al. [28] explicitly examined the incidence of side effect of a group of 89 healthy participants who experienced the GX platform. The occurrence of the side effects was very low, and no participants requested to terminate their participation in the study. To date, evidence from a fewer number of patient subjects with spinal cord injury (SCI) or stroke indicates that they also are not disturbed by side effects when using video-capture VR [25,34].

### Presence and enjoyment

Several studies examined the influence of video capture platform of the user's sense of presence and level of enjoyment. Rand et al. [28] in their study of 40 healthy young adult participants, compared two different VR platforms, the GX-monitor and a combination of GX environments viewed via an HMD. They found that the participants' sense of presence was significantly higher when using the GX monitor platform than when using the GX-HMD. In a companion study, which compared the GX-monitor with an HMD with two age groups, 33 young adults and 16 elderly participants, the older group felt a significantly higher sense of presence and enjoyment than did the younger group using the HMD. Lott et al. [37] used the IREX video capture platform and an HMD and found that the levels of presence reported by the young adult participants did not differ significantly for the two virtual reality conditions.

The results of these studies showed that a high sense of presence and level of enjoyment can be achieved in a video capture VR platform. They also demonstrate that user characteristics such as age influence the sense of presence.

In another study, Rand et al. [38] compared the sense of presence, performance and perceived exertion experienced by 30 healthy young participants when they engaged in two games performed within video-projected virtual environments that differed in their level of structure and spontaneity. The non-structured application was applied using VividGroup's Gesture Xtreme (GX) VR platform, and the structured application was applied using the IREX platform, a rehabilitation-oriented application of GX, developed to train a specific movement (e.g., shoulder abduction) in order to increase range of motion or endurance. No main effect or interaction effect was found for the sense of presence (assessed using Witmer & Singer's [39] Presence Questionnaire (PQ) although significant differences were found for several of the PQ sub-scales. It was concluded that it is possible to provide users with a satisfactory level of presence and enjoyment using both structured and non-structured paradigms. Therefore, both movement options, structured and non-structured, enhance the therapist's repertoire of VR intervention tools in order to maximize rehabilitation.

Rand at al. [40] reported the results of another study, in which two different video-capture platforms, GX and EyeToy, were compared to determine their effect on users' sense of presence, level of enjoyment, perceived exertion and side effects. In this study, 18 healthy young adults experienced two games in each platform (Birds & Balls and Soccer in GX and Kung-Foo and Wishy-Washy in EyeToy) in a counter-balanced order. There was no significant difference in the sense of presence between the two platforms. However, the EyeToy Kung-Foo game, which encourages participants to eliminate successive invading warriors by hitting at them, was found to be significantly more enjoyable than the other games. In a continuation of this study, Rand et al. [40] examined the feasibility of using the EyeToy with healthy elderly users. Ten healthy elderly participants, aged 59 to 80 years, found this platform easy to operate and enjoyable. The results for patients with stroke at a chronic stage (1–5 years post stroke) were similar to the healthy elderly. They thought that it could contribute to their rehabilitation process, and were able to operate the platform independently. The responses of a third group of users, patients with stroke at an acute stage (1–3 months post stroke), were somewhat different. They also reported that they enjoyed the experience; however, they became frustrated while performing the EyeToy games, even when played at the easiest levels. This latter observation highlights a major limitation of the closed architecture of the EyeToy; to date, Sony has been unwilling to adapt the games to include a greater range of levels of difficulty, nor to provide tools to external programmers to do so (R. Marks, personal communication). It also emphasized the effect that user characteristics, in this case, time post onset of stroke, have on the sense of presence.

The GX VR platform has consistently generated high levels of presence and enjoyment across a wide range of clinical populations and ages including adults with paraplegic spinal cord injury [34], stroke [25,33], and young adults with cerebral palsy and intellectual impairment [41]. A pilot study using the GX platform to determine its suitability for leisure time activities among older stroke survivors was carried out. These participants enjoyed the experience, and perceived it to be therapeutic [42].

### Performance outcomes and sensitivity of video capture VR

The measures of performance used by video-capture VR studies to date include response times to presented virtual stimuli, percent success with which a given game is performed (e.g., how many balls are repelled by the user in the role of soccer game goal keeper), a subjective report on how much effort the user has felt while in the environment. The chroma key video capture platforms such as GX and IREX also provide a relatively gross measure of limb kinematics. Whether these data have sufficient precision and resolution to warrant their inclusion in a research study remains to be investigated (F. MacDougal, personal communication).

Sveistrup, McComas and colleagues have used the IREX platform for balance retraining. Following six weeks of training at an intensity of three sessions per week, improvement was found for all 14 participants in both the VR and control groups [35]. However, the VR group reported more confidence in their ability to "not fall" and to "not shuffle while walking". The same research group has also demonstrated that an exercise program delivered via video capture VR can improve balance and mobility in adults with traumatic brain injury [43] and the elderly [44].

Kizony et al. [34] performed a feasibility study of the GX-VR platform to train balance of people who had a paraplegic SCI. The study included 13 adult participants who had paraplegia. Results from the patient group were compared to data from a parallel study of a group of 12 healthy adult participants who performed a similar protocol, while sitting on a chair with hands supported. The results showed that the participants with SCI who had better balance function performed higher within the virtual environments and the healthy participants performed significantly better than the participants with paraplegia. This platform appeared to be suitable for use with people who have paraplegia and it was able to differentiate between participants with different levels of balance function.

In a second study Kizony et al. [25] examined the relationships between cognitive and motor ability and performance within the GX-virtual environments with people who have had a stroke. Thirteen older adult patients with stroke participated in the full study. Significant moderate positive correlations were found between VR performance and cognitive abilities suggesting that higher cognitive abilities relate to higher performance within the VR. In contrast, almost no positive correlations were found with the motor abilities. Indeed, as pointed out by these authors, perhaps motor performance demands and their characteristics should not be expected to be identical within the real and the virtual worlds. It may be that differences in presence, motivation, or other factors influence the movement patterns differently in virtual versus natural environments. This result is in accordance with Lott et al.'s [38] findings which showed significant differences between functional lateral reach performed in a real versus virtual environment. They reported that the participants reached significantly further when virtual objects were presented within the virtual environment using a video capture VR platform than when they were asked to touch a person hand standing on their side. They suggest that embedding the reaching task in a game shifts the person's attention from the possibility of losing his balance thereby enabling him to achieve greater function.

Rand et al. [28] used a virtual office environment which was developed by Rizzo et al., [15] and was displayed both via an HMD and via the GX-monitor platform. In this case, participants stood in front of the GX monitor and visually scanned the Virtual Office. Performance by both age groups was significantly higher when using the GX-monitor platform than when using an HMD, whereas the younger group's visual scan ability was better than the elderly on both platforms. The results also demonstrated the effect that different user characteristics, such as age and gender, have on the VR experience and thus should be taken into consideration when considering which VR platform to use in rehabilitation.

Weiss at al. [41], in a study of five young male adults with physical and intellectual disabilities, explored ways in which virtual reality could provide positive and enjoyable leisure experiences during physical interactions with different game-like virtual environments and potentially lead to increased self-esteem and a sense of self-empowerment. The results of this study showed that the GX-VR platform was feasible for use with this population. The participants were able to use the platform and expressed their considerable enjoyment from the virtual games. However, the authors raised several concerns, especially that some of the participants displayed involuntary movement synergies, increased reflexes and maladaptive postures due to the too difficult levels of the games that were used in study. Thus, a more controlled study with the same population is currently in progress in order to examine more thoroughly the potential of the platform as a mean for providing leisure opportunities to this population.

Performance within two games (Kung-Foo and Wishy-Washy) was measured while three different groups, young adult participants, healthy senior participants and individuals who were several years post-stroke, used several of the EyeToy games [40]. Performance was scored for each game in terms of how much of a given activity (e.g., how many windows washed, how many warriers eliminated) was accomplished within a preset time limit. Higher scores were achieved when clients were able to perform these activities faster and/or more accurately. There were significant differences in performance between the young and stroke groups, with the young adults having greater success in both games than the stroke group. The older adult group performed as well as the younger group.

The performance results described above highlight the interplay between the user and VR platform characteristics, and emphasize the importance of taking these characteristics into consideration while using VR in rehabilitation. Moreover, they demonstrate the sensitivity of the VR performance measures in their capacity to differentiate between levels of participant ability.

Due to the motivating nature of the game-like environments, it is important to determine how much effort healthy subjects and those with disabilities expend while engaged in these tasks. In a study of healthy young adults, the participants using the GX platform perceived the highest level of exertion while playing Soccer, less for Birds & Balls and still less for a third game, Snowboard where only weight transfer was needed [28]. When differences between the age groups were assessed, the younger group perceived higher levels of exertion in comparison to the older group. There were also differences in the perceived level of exertion of the Birds & Balls game in GX as compared to comparable games in the EyeToy [40]. Overall, the level of perceived exertion was rated as "somewhat difficult" which is an ideal level to use in therapy.

### Initial comparisons of VR-based intervention to conventional therapy

Using the IREX platform, Sveistrup et al. [35] performed two studies designed to compare VR-delivered therapy to conventional therapy. In their first study, patients suffering from frozen shoulder received exercise either via IREX applications or via conventional physiotherapy. In both cases, therapy was directed at improving the quality of three specific shoulder joint movements. In the second study, individuals who suffered from post-traumatic brain injury were assigned to either VR-based (applications such as the virtual soccer game were used where patients were encouraged to reach towards the virtual stimulus in addition to weight transfer) or conventional therapy (e.g., stepping, picking up objects, reaching) for balance training for a total of 24 sessions. In their report on preliminary data from 14 patients, the authors concluded that both exercise programs resulted in improvement of patients' balance. However, additional benefits were identified for the VR group, including greater enthusiasm for the VR-delivered therapy program, increased enjoyment while doing the exercises, improved confidence while walking and fewer incidents of falling.

Cunningham & Krishack [32] presented VR as it was used in occupational therapy to improve balance and dynamic standing tolerance with geriatric patients. They reported greater improvement in dynamic standing tolerance in a small group of older adults following a VR therapy than in a small group following a standard occupational therapy. More recently, Bisson, et al. [44] demonstrated significant improvements in balance and functional mobility in community-living older adults following a VR exercise program delivered with the IREX platform. The comparison group completed a biofeedback exercise program and also demonstrated significant balance improvement.

Analysis of conventional and video capture VR treatment for SCI by specialists in rehabilitation highlighted several key differences between the two methods of intervention [34]. First, control over delivery of the stimuli via the VR platform enabled the therapist to intervene more effectively, especially in terms of physical guidance and support. In addition, the VR platform allowed precise control over delivery of the number of stimuli simultaneously presented to the patient as well as their speed and direction. These features appeared to increase the number of times a desired balance-recovery movement was performed by patients. Finally, the ease with which this platform elicited dynamic equilibrium recovery responses, an essential component in balance training and encouraged weight transfer movements was remarkable. In contrast, the static presentation of stimuli during conventional therapy restricts intervention to focus almost exclusively on weight transfer.

### Towards functional video-capture environments

**Figure 3:**
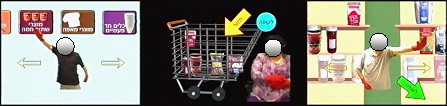
Screen shots of the VMall showing clients with stroke selecting a shopping aisle (left panel), a food item (middle panel) and verifying the contents of the shopping cart (right panel).

One of the newest developments in video-capture VR is the simulation of more functional environments. Rand et al. [45] have created a Virtual Mall (VMall), using the GX platform. It has been designed to support intervention of patients following a stroke who have motor and/or executive functions deficits that restrict their everyday activities. This environment enables participants to engage in tasks based on typical daily activities such as shopping in a supermarket. In the initial application, shown in Figure 3, the user moves from aisle to aisle by activating icons located on a large monitor around thereby encouraging active movement, transfer of weight from side to side, and balance reactions. Virtual food items are manipulated (e.g., selected from a shelf and placed in a supermarket cart in accordance with a shopping list selected in advance. The performance of the task provides multiple opportunities to make decisions, plan strategies and multitask, all in a relatively intuitive manner. Output measures include a record how well the user accomplishes the task (e.g., how many correct items selected) will be recorded and saved thus giving an option to monitor improvement over time. Initial performance measures and user feedback has been recorded from six patients who had a stroke more than two years since onset and suffer from residual motor and cognitive deficits. The results suggest that the VMall provides a motivating task that requires active movement as well as the ability to plan and problem solve.

Sony's EyeToy Wishy Washy application involves the cleaning of successive dirty windows via wiping movements of the hand and arms. Most recently, VividGroup has developed a laundry application (V.J. Vincent, personal communication). These moves towards more functional applications are encouraging.

## Conclusions

Evidence from the literature has demonstrated the feasibility, usability and flexibility of video-capture VR, and there is little doubt that this technology provides a useful tool for rehabilitation intervention. The results of presence questionnaires, reports of user satisfaction, and the sensitivity to differences in user ability as functions of age, gender and disability are all strong indicators of the suitability of this tool. A short video-clip, taken from a local news report of applications of video-capture VR for stroke, illustrates the extremely positive response of one user to the use of this technology (see Video 1).

To date, as indicated by the studies reviewed above, video capture VR shows great promise for a variety of therapeutic goals including intervention for cognitive and motor rehabilitation, functional activities and leisure opportunities. The general assets of virtual reality summarized above combined with several assets that are unique to video-capture VR, are compelling arguments for the inclusion of this technology in the repertoire of tools available in clinical settings.

Market demand, user interest and improvements in technology have led to the availability of a number of different video-capture platforms. There is no doubt that these platforms are valuable as intervention tools during the rehabilitation of patients with neurological and musculoskeletal disorders. Motivated patients would be encouraged to practice movements in a repetitive manner thereby improving their condition, an achievement that is not easy to attain via conventional therapy [46]. Currently, the two main contenders for the rehabilitation market are VividGroup's GX and IREX platforms and Sony's PlayStation II's EyeToy. Both use large monitors to display real-time images of users interacting with virtual objects in a simulated environment. The VividGroup platforms are considerably more expensive and require a more elaborate setup including a chroma key blue/green backdrop behind the user and bright, ambient lighting. Sony's EyeToy is an off-the-shelf, low-cost gaming application that may be run under almost any ambient conditions.

Studies comparing these two platforms have shown that presence, enjoyment, usability and performance were equivalent under many conditions and for diverse users. Thus, despite the EyeToy's limitations, its low cost, user-friendly interface and simple setup requirements makes it highly attractive to therapists. It may be readily acquired for use in any clinical setting, and even be purchased for use at home to provide regular, intensive therapy after discharge from hospital.

Nevertheless, it is clear that the EyeToy is not suited for use with the most severely impaired users. The currently available games seem to have a broad appeal for users of different ages but an open architecture that permits adaptations of existing applications and development of new environments appears to be a basic requirement to make this platform truly functional as a clinical tool. A system for generating an outcomes report comparable to the IREX platform would also be of great benefit for clinicians. Additional low-cost video-capture platforms are currently under development (M. Shahar, personal communication). Moreover, video-capture platforms that will provide three dimensional, functional environments will likely soon become available (Cohen and Rizzo, personal communication).

In contrast to the EyeToy's closed architecture, VividGroup's IREX platform provides a user-friendly interface that a therapist may use to specify a much greater range of levels of difficulty. Their SDK (Software Development Kit) provides programmers with the ability to further adapt existing applications such as the standard set of games [33] and to design and implement novel applications such as the virtual mall described above [45]. The popular press has been generating a considerable amount of publicity in the EyeToy platform [31], and it is clear that low-cost video-capture systems such as these are poised to make VR available to a wide range of users. We anticipate that future developments in technology, such as low-cost virtual environments that are more functional will enable clinicians to take advantage of the considerable benefits that VR has for rehabilitation.

## Supplementary Material

Additional File 1 Video 1: This video clip shows a patient who had a stroke using the VividGroup VR system for cognitive and motor rehabilitation. Click here for file
